# Nanomaterials and Nanotechnology in Wastewater Treatment

**DOI:** 10.3390/nano11061539

**Published:** 2021-06-10

**Authors:** George Z. Kyzas, Athanasios C. Mitropoulos

**Affiliations:** Department of Chemistry, International Hellenic University, 65404 Kavala, Greece

The rapidly increasing population, depleting water resources, and climate change resulting in prolonged droughts and floods have rendered drinking water as a competitive resource in many parts of the world. Therefore, any form of water reuse or recycle will help to mitigate this challenge. The careful management of water and wastewater is a big challenge and the “hot” trend of recent research. During the past century, a huge amount of wastewater was discharged into rivers, lakes, and coastal areas. This resulted in serious pollution problems in the aqueous environment. Municipal, industrial, and natural activities produce large quantities of liquid wastes and effluents which pose severe threats to the environment and human health. So, it is mandatory to find the appropriate technique in order to efficiently treat and manage water and wastewaters. Some indicative/conventional methods are biological treatments, adsorption, flocculation, oxidation, membranes, filtration, etc. These conventional technologies focus only on the primary wastewater treatment, especially on the physical separation of solid particles and the release of high concentrations of toxic phosphorus, nitrogen, and other ionic compounds into the environment. Thus, the latest technology involving nanotechnology is highly potent in advancing wastewater treatment via nanomaterials (nanoadsorbents, nanocomposites, (photo)catalysts, nanofiltration, nanomembranes, nanoparticles, etc.). These nanomaterials have been established in the development of separation membranes, catalysts, and adsorbent materials to enhance the removal of specific components of wastewater and improve productivity. Zero-valent metal nanoparticles (Ag, Fe, and Zn), metal oxide nanoparticles (TiO_2_, ZnO, and iron oxides), carbon nanotubes (CNTs), nanocomposites, and many other types of nanomaterials are already used in wastewater treatment. All of the above can be achieved by using nanotechnology. This Special Issue on “Nanomaterials and Nanotechnology in Wastewater Treatment” seeks high-quality works and topics (not only those) focusing on the latest approaches based on nanotechnology to efficiently treat wastewater.

This Special Issue (belongs to the section Environmental Nanoscience and Nanotechnology) on “Nanomaterials and Nanotechnology in Wastewater Treatment”, we believe, succeeded to present such high-quality works and topics focusing on the latest novel nanotechnology works on wastewater processes. This Special Issue consists of 21 works (19 research articles, 1 review paper, and 1 communication) from distinguished authors worldwide [1,2,3,4,5,6,7,8,9,10,11,12,13,14,15,16,17,18,19,20,21].

Khan et al. [7] evaluated the Fe–Mg binary oxide for As(III) adsorption in batch mode. Detailed synthesis, characterization and kinetic modeling were presented. Yadav et al. [19] studied the synthesis and characterization of methionine-functionalized graphene oxide/sodium alginate biopolymer nanocomposite hydrogel beads and their application as adsorbents for the removal of fluoroquinolone antibiotics (isotherms and kinetics were analyzed in detail). In another interesting work [5], authors investigated the CO_2_/CH_4_ and He/N_2_ separation properties and water permeability valuation of mixed matrix MWCNTs-based cellulose acetate flat sheet membranes in a study about the optimization of the filler material dispersion method. Zhu et al. [21] studied the adsorption kinetics of arsenic (V) on nanoscale zero-valent iron supported by activated carbon, while Ramos-Guivar et al. [13] focused on the improved removal capacity and equilibrium time of maghemite nanoparticles growth in zeolite type 5A for Pb (II) adsorption. In another “green” nanoadsorption study, Das et al. [3] investigated the green synthesis, characterization, and application of natural product coated magnetite nanoparticles for wastewater treatment (the effect of synthesized magnetic nanoparticles in wastewater treatment (bacterial portion), dye adsorption, toxic metal removal, as well as antibacterial, antioxidant, and cytotoxic activities were studied). Sekar et al. [14] published a work about the upcycling of wastewater via effective photocatalytic hydrogen production, using MnO_2_ nanoparticles decorated activated carbon nanoflakes, while another study published by Mashentseva et al. [11] focused on Cu/CuO composite track-etched membranes for catalytic decomposition of nitrophenols and application to the removal of As(III). Yadav et al. [20] studied the synthesis and characterization of amorphous iron oxide nanoparticles by the sonochemical method and their application for the remediation of heavy metals (lead, chromium) from wastewater. Tao et al. [17] published the aerobic oil-phase cyclic magnetic adsorption to synthesize 1D Fe_2_O_3_@TiO_2_ nanotube composites for enhanced visible-light photocatalytic degradation, while Ahmadi et al. [1] studied the acid dye removal from aqueous solution by using neodymium(III) oxide nanoadsorbents. Shu et al. [15] used almond shell-derived, biochar-supported, nano-zero-valent iron composite to remove Cr(VI) from aqueous solutions. Hasan et al. [6] synthesized in situ copolymerized polyacrylamide cellulose supported Fe_3_O_4_ magnetic nanocomposites to adsorptively remove Pb(II), with special focus on artificial neural network modeling. On the other hand, Kumar et al. [16] studied the silver quantum dot decorated 2D-SnO_2_ nanoflakes for photocatalytic degradation of the water pollutant Rhodamine B, while Xia et al. [18] investigated the removal of Hg(II) by EDTA-functionalized magnetic CoFe_2_O_4_@SiO_2_ nanomaterial with core-shell structure. The enhanced kinetic removal of Ciprofloxacin onto metal–organic frameworks by sonication, process optimization and metal leaching study was published by Dehghan et al. [4], and Lee et al. [8] published the continuous flow removal of anionic dyes (Evans blue) in water by chitosan-functionalized iron oxide nanoparticles incorporated in a dextran gel column. Li et al. [9] investigated the synthesis of hierarchical porous carbon in molten salt and its application for methylene blue and methyl orange adsorption. Pruna et al. [12] tailored the performance of graphene aerogels for oil/organic solvent separation by one-step solvothermal approach, while Lin et al. [10] occupied with the preparation of CoMn_2_O_4_ catalyst by using the sol–gel method for the activation of peroxymonosulfate and degradation of UV filter 2-phenylbenzimidazole-5-sulfonic acid. Also, a review article was published and included in the SI about the recent progress in heavy metal ion decontamination based on metal–organic frameworks [2].

Many authors, whom we, as editors, thank very much, from various countries contributed marvellously to the present Special Issue. All the aforementioned topics and many more were explored in detail. Certainly, the field of wastewater treatment using nanomaterials and generally nanotechnology is vast; the present study hopefully adds one more useful contribution.
